# MRI Visual Ratings of Brain Atrophy and White Matter Hyperintensities across the Spectrum of Cognitive Decline Are Differently Affected by Age and Diagnosis

**DOI:** 10.3389/fnagi.2017.00117

**Published:** 2017-05-09

**Authors:** Hanneke F. M. Rhodius-Meester, Marije R. Benedictus, Mike P. Wattjes, Frederik Barkhof, Philip Scheltens, Majon Muller, Wiesje M. van der Flier

**Affiliations:** ^1^Department of Neurology, Alzheimer Center, VU University Medical Centre, Amsterdam NeuroscienceAmsterdam, Netherlands; ^2^Department of Radiology and Nuclear Medicine, VU University Medical Centre, Amsterdam NeuroscienceAmsterdam, Netherlands; ^3^Institutes of Neurology and Healthcare Engineering, UCLLondon, UK; ^4^Department of Internal Medicine, Section Geriatrics, VU University Medical CentreAmsterdam, Netherlands; ^5^Department of Epidemiology and Biostatistics, VU University Medical CentreAmsterdam, Netherlands

**Keywords:** Alzheimer's disease, mild cognitive impairment (MCI), MRI, prognosis, diagnostic test assessment

## Abstract

**Aim:** To assess the associations of age and diagnosis with visual ratings of medial temporal lobe atrophy (MTA), parietal atrophy (PA), global cortical atrophy (GCA), and white matter hyperintensities (WMH) and to investigate their clinical value in a large memory clinic cohort.

**Methods:** We included 2,934 patients (age 67 ± 9 years; 1,391 [47%] female; MMSE 24 ± 5) from the Amsterdam Dementia Cohort (1,347 dementia due to Alzheimer's disease [AD]; 681 mild cognitive impairment [MCI]; 906 controls with subjective cognitive decline). We analyzed the effect of age, APOE e4 and diagnosis on visual ratings using linear regression analyses. Subsequently, we compared diagnostic and predictive value in three age-groups (<65 years, 65–75 years, and >75 years).

**Results:** Linear regression analyses showed main effects of age and diagnosis and an interaction age^*^diagnosis for MTA, PA, and GCA. For MTA the interaction effect indicated steeper age effects in MCI and AD than in controls. PA and GCA increased with age in MCI and controls, while AD patients have a high score, regardless of age. For WMH we found a main effect of age, but not of diagnosis. For MTA, GCA and PA, diagnostic value was best in patients <65 years (optimal cut-off: ≥1). PA and GCA only discriminated in patients <65 years and MTA in patients <75 years. WMH did not discriminate at all. Taking into account APOE did not affect the identified optimal cut-offs. When we used these scales to predict progression in MCI using Cox proportional hazard models, only MTA (cut-off ≥2) had any predictive value, restricted to patients >75 years.

**Conclusion:** Visual ratings of atrophy and WMH were differently affected by age and diagnosis, requiring an age-specific approach in clinical practice. Their diagnostic value seems strongest in younger patients.

## Introduction

The current diagnostic criteria for mild cognitive impairment (MCI) and dementia due to Alzheimer's disease (AD) advise to apply biomarkers such as MRI features, to identify patients with (underlying) AD pathology (Dubois et al., 2007, 2014; Albert et al., 2011; McKhann et al., 2011). The criteria do not specify how MRI features should be measured, what cut-offs should be used and whether a patient's age should be taken into account (Frisoni et al., 2011). Studies demonstrating discriminatory value of atrophy, such as medial temporal lobe atrophy (MTA), parietal atrophy (PA) and global cortical atrophy (GCA) in AD, often use automatic quantitative MRI analysis (van de Pol et al., 2006; Sluimer et al., 2008; Henneman et al., 2009a; Trzepacz et al., 2014). However, these analyses are time consuming hence hard to apply in daily clinical practice. A feasible way of applying MRI features in daily practice is to use established visual rating scales for atrophy measures and vascular white matter changes (Scheltens et al., 1992, 1995; Wattjes et al., 2009).

The presence of MTA has been shown to differentiate patients with dementia due to AD from controls and to predict progression to dementia in MCI patients (Scheltens et al., 1992; Jack et al., 2002; Korf et al., 2004; Vos et al., 2012; Clerx et al., 2013; Ferreira et al., 2015). However, medial temporal lobe atrophy also occurs in normal aging (Jernigan et al., 2001; van de Pol et al., 2006; Barkhof et al., 2007). To discriminate both young and old controls from AD, an average score of the left and right sides of MTA ≥ 1 has been proposed for patients <75 years and MTA ≥ 1.5 for patients >75 years (Scheltens et al., 1992, 1995; Schoonenboom et al., 2008). Recently two studies, based on the same cohort, have suggested to increase the cut-off for patients <75 years to MTA ≥ 1.5, for patients >75 years to MTA ≥ 2 and to add a specific cut-off of MTA ≥ 2.5 for patients aged >85 years (Pereira et al., 2014; Ferreira et al., 2015). Since these studies used patients with a mean age of 75, it remains uncertain what the optimal cut-off in younger patients would be.

In younger patients, PA is increasingly recognized as an important feature of AD (Koedam et al., 2010). Rating PA improves the distinction of early onset AD patients from younger controls, but seems to be less suited to separate older AD patients from older controls (Lehmann et al., 2012; O'Donovan et al., 2013). No age-specific cut-offs have yet been suggested (Koedam et al., 2011; Ferreira et al., 2015). Only one study assessed the diagnostic value of combining MTA with PA, but this study did not take age into account (Ferreira et al., 2015). Being affected by parietal atrophy as well, the GCA scale has a lot of overlap with the PA scale. However, no cut-offs for the use of this scale as a diagnostic or predictive marker exist (Pasquier et al., 1996; Scheltens et al., 1997; Henneman et al., 2009b; Fjell et al., 2013).

Particularly in older patients, dementia pathology is often mixed including neurodegenerative and vascular changes. Therefore, in addition to measures of atrophy, it is common practice to estimate the extent of small vessel disease (SVD), such as white matter hyperintensities (WMH) in the diagnostic workup (van der Flier et al., 2004; Kester et al., 2014). A recent CT study showed an unexpected low percentage of WMH in elderly patients (Claus et al., 2015). It has been suggested that WMH may predict progression in the MCI stage, but other studies have found no such effect (Prins et al., 2013; Mortamais et al., 2014). The WMH severity can be rated using Fazekas' scale, but optimal cut-offs for separating controls from AD taking into account age have not been reported (Fazekas et al., 1987). Details regarding the afore mentioned scales can be found in Table 1.

**Table 1:**
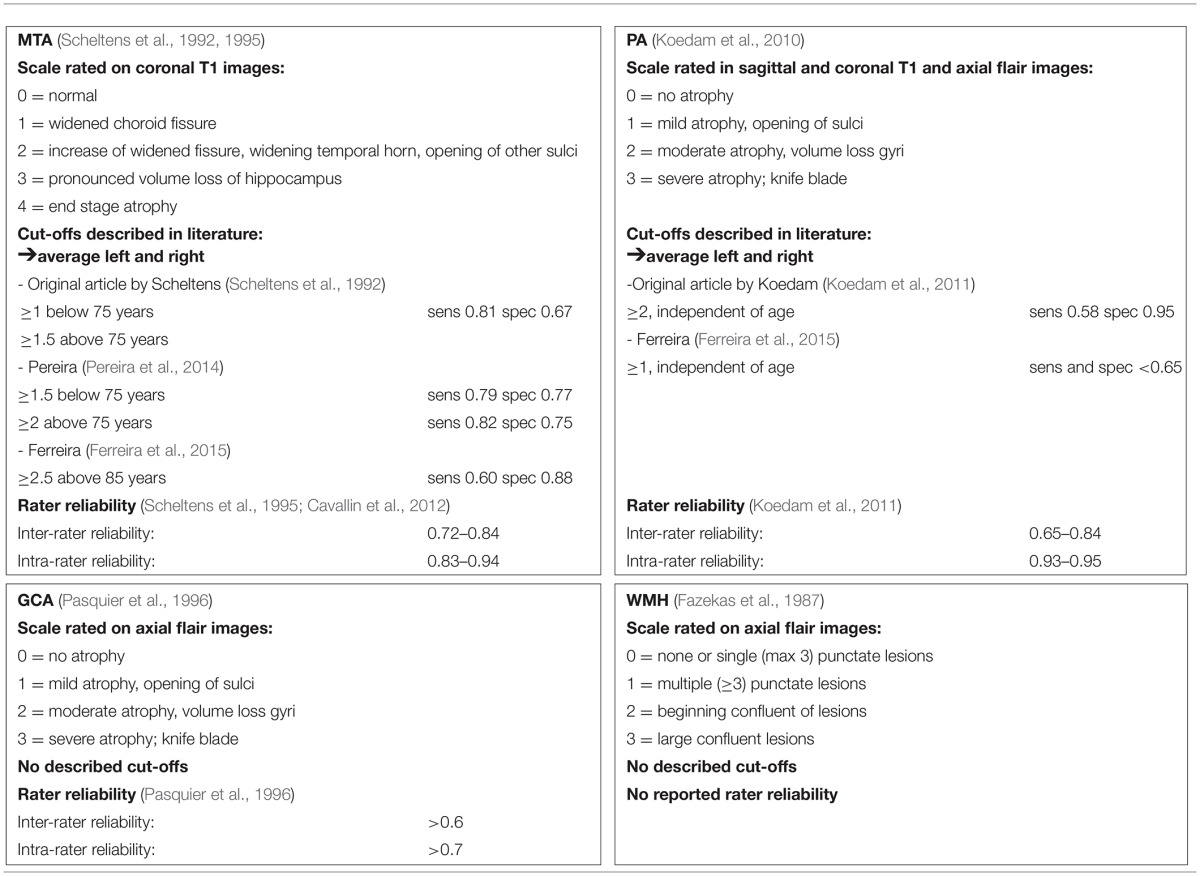
**Details on used visual ratings scale of MTA, PA, CGA, and WMH**.

*Sens, sensitivity; spec, specificity. Inter-rater and intra-rater reliability is presented as Cohen weighted Kappa*.

The aim of our study was to explore the effect of age on the diagnostic value of visual ratings of MTA, PA, GCA, and WMH for discriminating controls from AD and for predicting progression to dementia in MCI in a very large memory clinic cohort (van der Flier et al., 2014). Second, we evaluated the effect of APOE genotype. Our ultimate goal is to provide practical support to clinicians to improve the effective incorporation of MRI visual ratings scale in daily practice.

## Methods

### Subjects

We included 2,934 patients from the Amsterdam Dementia Cohort who had visited the Alzheimer center between 2000 and 2015 (van der Flier et al., 2014). Of these patients, 906 were diagnosed with subjective cognitive decline (SCD), who served as controls, 681 with MCI and 1,347 with AD. Subjects were included if MRI and mini mental state examination (MMSE; Folstein et al., 1975), performed within 6 months of baseline diagnosis, were available. The local medical ethical committee approved the study, according to the declaration of Helsinki. All patients provided written informed consent for their clinical data to be used for research purposes.

### Clinical assessment

At baseline, patients received a standardized and multi-disciplinary work-up, including medical history, physical, neurological and neuropsychological examination, MRI and laboratory tests. Cognitive functions are assessed with a standardized test battery, including the MMSE and Cambridge cognitive examination for global cognitive decline (Folstein et al., 1975; Derix et al., 1991). For memory we use the visual association test (VAT) and Rey auditory verbal learning task (Saan and Deelman, 1986; Lindeboom et al., 2002). For language we use VAT naming and category fluency (Lindeboom et al., 2002; Van der Elst et al., 2006). For attention and executive functions we use the trail making test A and B and the digit span (Reitan, 1958; Lindeboom and Matto, 1994). More details can be found in our cohort paper (van der Flier et al., 2014). Diagnoses were made in a multidisciplinary consensus meeting (van der Flier et al., 2014). Patients were labeled as SCD when the cognitive complaints could not be confirmed by cognitive testing and criteria for MCI, dementia or any other neurological or psychiatric disorder known to cause cognitive complaints were not met. MCI was diagnosed using Petersen's criteria; in addition all patients fulfilled the core clinical criteria of the NIA-AA guidelines for MCI (Petersen, 2004; Albert et al., 2011). Patients were diagnosed with probable AD using the criteria of the National Institute for Neurological and Communicative Diseases Alzheimer's Disease and Related Disorders Association; all patients also met the core clinical criteria of the National Institute on Aging-Alzheimer's Association guidelines for AD (McKhann et al., 1984, 2011).

### Follow-up

Follow-up for MCI patients took place by annual routine visits to our memory clinic in which patient history, cognitive tests, and a physical and neurologic examination were repeated. Follow-up data were available in 464(68%) MCI patients, with a mean duration of follow-up of 2.5 ± 1.7 years. Of these patients, 255(55%) remained stable, 161(35%) progressed to AD and 48(10%) progressed to another type of dementia.

### MRI

Subjects were scanned with a standardized scan protocol on 1.0 T, 1.5 T, and 3.0 T whole body MRI systems as part of their diagnostic work-up. Over time, the core protocol remained comparable and always included 3DT1 with coronal slices and FLAIR with axial slices. Details on acquisition parameters per scanner can be found in Supplementary Table 1. All scans were visually rated by a trained rater after they had completed the required training and obtained a weighted kappa of at least 0.80 for MTA, 0.60 for GCA, and 0.70 for Fazekas, and subsequently evaluated in a consensus meeting with our experienced neuroradiologist. The raters were blinded for diagnosis. Visual rating of MTA was performed on oblique coronal T1-weighted images according to the 5-point (range 0–4) Scheltens scale from the average score of the left and right sides (Scheltens et al., 1992, 1995). PA was rated using the posterior cortical atrophy scale (range 0–3), using T1 and FLAIR weighted images viewed in sagittal, axial and coronal planes, computing an average score of the left and right sides (Koedam et al., 2010, 2011; Lehmann et al., 2013). Global cortical atrophy (GCA) was assessed visually on axial FLAIR images (range 0–3) (Pasquier et al., 1996). The degree of white matter hyperintensities severity was rated on axial FLAIR images using Fazekas' scale (range 0–3) (Fazekas et al., 1987). More details can be found in Table 1.

### APOE genotyping

DNA was isolated from 10 ml of EDTA blood. APOE genotype was determined with the light cycler APOE mutation detection method (Roche diagnostics GmbH, Mannheim, Germany). According to APOE e4 status, patients were dichotomized into carriers (hetero- and homozygous) and non-carriers. APOE status was available for 2410(82%) subjects.

### Statistical analyses

For statistical analyses, we used SPSS version 20 (IBM, Armonk, NY, USA). We compared visual ratings according to the baseline diagnosis (controls, MCI and AD) using Kruskal-Wallis tests and *post-hoc* Mann-Whitney U-tests. We used Spearman's correlations to assess correlations between visual rating scales.

We used linear regression analyses to assess the combined effect of age and diagnosis on visual ratings (using separate models for each rating scale). As independent variables we entered diagnosis (using dummy variables), age (continuous) and the interaction terms for age^*^diagnosis. In a second model we additionally added APOE (dichotomized) as independent variable and the interaction term age^*^APOE. To confirm the age effect on visual ratings we repeated the linear regression analyses entering as independent variable, instead of diagnosis, MMSE (continuous) and the interaction term for age^*^MMSE. To allow comparison of the different models, we report standardized betas (st beta).

Subsequently, we created three age strata (<65 years, 65–75 years and >75 years) and evaluated the diagnostic ability of each visual rating scale to separate patients with dementia due to AD from controls per age group. Sensitivity, specificity, positive predictive value (PPV) and negative predictive value (NPV) and the Youden index [(sensitivity + specificity)-1] (Youden, 1950) were calculated for different cut-off points in the three age groups using cross tabulation. When we repeated the linear regression analyses adding APOE, we found only an effect of APOE e4 presence on the MTA scale. Therefore, we repeated the evaluation of diagnostic ability for MTA only, stratifying for APOE e4 carriers (controls vs. AD) and APOE e4 non-carriers (controls vs. AD), excluding 524(18%) of subjects in which APOE was not available. The highest Youden index indicated the optimal cut-point, we took a Youden index >0.50 as a minimum. For the scales showing a Youden index >0.50, we assessed the effect of combining the scales at their optimal cut-off. We created a new variable consisting of 4 levels: 1. normal MTA and normal PA (reference group), 2. normal MTA and abnormal PA, 3. abnormal MTA and normal PA, 4. abnormal MTA and abnormal PA. This was also done for the combination of MTA and GCA.

Finally, we assessed the predictive value of the visual ratings for dementia due to AD in MCI patients, stratified by age group. We used Cox proportional hazard models, taking into account variability in time to follow up. Baseline MTA, PA, CGA, and WMH were entered dichotomized, in separate models, at the earlier derived optimal, age-specific cut-offs and, in addition as continuous values. In a separate model, we evaluated the combined effect of MTA and PA and of MTA and GCA using the newly constructed 4 level variables, as described above. Event variable was progression to dementia due to AD, excluding subjects with progression to another type of dementia, and in another model progression to all types of dementia. Sex was entered as co-variate. HR with 95% confidence intervals (CI) are presented.

A *p* < 0.05 was considered significant. Since we focus on discriminatory and predictive value, rather than statistical significance, we did not adjust for multiple comparisons.

## Results

### Baseline characteristics

Table 2 shows the baseline characteristics of the total population. Patients with MCI and AD were older and had more WMH than controls. Patients with AD were more often female, more often APOE e4 carrier, had the lowest MMSE score and highest MTA, PA, and GCA compared to controls and MCI. When we assessed correlations between the visual rating scales using Spearman's rho, we found the strongest correlation between PA and GCA (*r* = 0.732) and the weakest correlation for WMH and PA (*r* = 0.133; Supplementary Table 2).

**Table 2:** **Baseline characteristics of controls, MCI and AD patients in the total group**.

	**Control**	**MCI**	**AD**
N	906	681	1347
Age	62 ± 9	69 ± 9	69 ± 9^a^
Female^#^	404 (45%)	271 (40%)	716 (53%)^a^^b^
MMSE	28 ± 2	26 ± 2	20 ± 5^a^^b^
Level of education	5 ± 1	5 ± 1	5 ± 1^a^
APOE e4 carrier^#^	281 (36%)	285 (54%)	729 (54%)^a^^b^
MTA	0.4 ± 0.6	1.0 ± 0.9	1.6 ± 0.9^a^^b^
PA	0.6 ± 0.7	0.9 ± 0.7	1.4 ± 0.7^a^^b^
GCA	0.4 ± 0.6	0.8 ± 0.7	1.2 ± 0.7^a^^b^
WMH	0.7 ± 0.7	1.1 ± 0.9	1.1 ± 0.9^a^

Values are mean ± standard deviation or n (%). Group differences between the different diagnostic groups were estimated using Chi-quadrate test^#^ and Kruskal-Wallis test and post hoc Mann-Whitney U-tests when appropriate. Please note that although we report mean ± standard deviation for the visual rating scales, we used non-parametric tests.

a P < 0.05 compared to control,

b *P < 0.05 compared to MCI*.

### Influence of age and diagnosis on visual ratings

**Figure 1:**
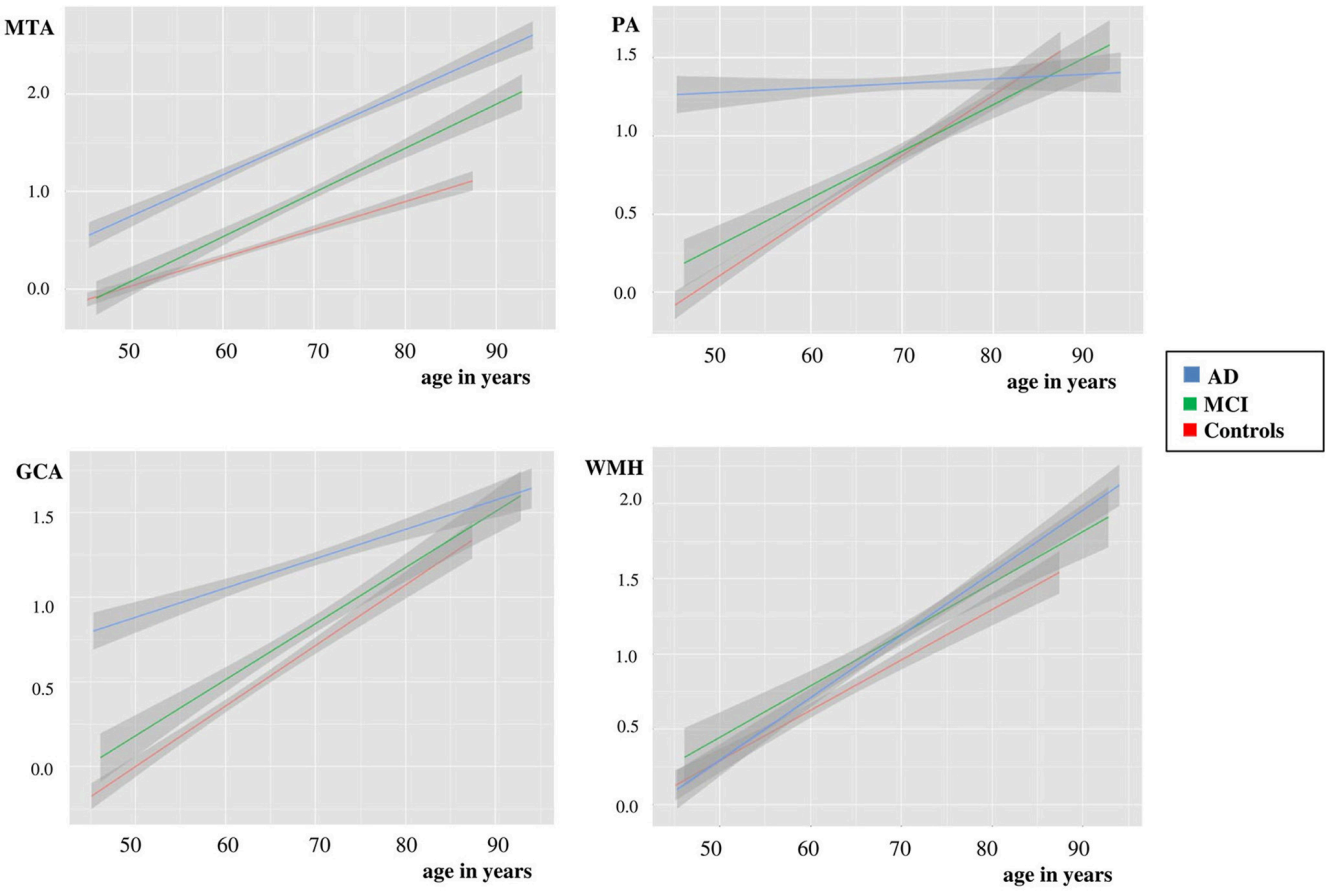
**Plots using linear regression analyses of MTA, PA, GCA, and WMH vs. age in controls, MCI and dementia due to AD, after correction for gender**. Y-as: respectively mean MTA (left + right/2), PA (left + right/2), GCA, and WMH, X-as: age in years, 95% confidence interval is presented by the gray area on both sides of each line.

We used linear regression analyses to assess the combined effect of age and diagnosis on each visual rating (Figure 1 and Table 3). For MTA we found main effects of age and diagnosis. In addition there was an interaction effect for age^*^diagnosis, indicating a somewhat steeper age effect in patients with MCI and AD than in controls. For PA and GCA, we found main effects of age and diagnosis. In addition, there was an interaction effect for AD age^*^diagnosis, indicating that AD patients have a higher score, regardless of their age, while in MCI and controls, PA and GCA increased with age. For WMH we only found a main effect of age but no main effect of diagnosis nor interaction between age and diagnosis. When we added APOE and age^*^APOE to the model, we found a main effect of APOE on MTA indicating more MTA in case of APOE e4 presence, and an interaction effect of age^*^APOE, indicating a steeper age effect in APOE non-carriers on MTA. However, in PA, GCA, and WMH we found no main effect of APOE nor an interaction effect of age^*^APOE.

**Table 3:** **Combined effect of age and diagnosis on visual ratings**.

	**MTA**		**PA**		**GCA**		**WMH**	
	**St beta**	***p***	**St beta**	***p***	**St beta**	***p***	**St beta**	***p***
Constant	−1.55	<0.001	−1.94	<0.001	−1.86	<0.001	−1.33	<0.001
Sex, male	0.07	<0.001	0.08	<0.001	0.08	<0.001	−0.06	<0.001
Age	0.30	<0.001	0.46	<0.001	0.45	<0.001	0.37	<0.001
**DIAGNOSIS**								
MCI	−0.32	<0.001	0.39	005	0.18	<0.001	0.11	0.443
AD	0.07	0.563	1.78	<0.001	1.11	<0.001	−0.17	0.227
**INTERACTION**								
Age^*^MCI	0.49	<0.001	−0.36	0.014	−0.09	0.508	−0.02	0.912
Age^*^AD	0.45	<0.001	−1.48	<0.001	−0.75	<0.001	0.26	0.081
*R* square	0.43		0.27		0.33		0.19	
F	367.98		183.04		236.90		116.93	
df regression	6		6		6		6	
df residual	2,927		2,926		2,927		2,927	

Linear regression analyses were used, using separate models for each rating scale. As independent variables we entered diagnosis (using dummy variables), age (continuous) and the interaction terms for age^*^diagnosis. St beta: standardized coefficients beta, p, p-value; F, Fisher; df, degrees of freedom.

When we repeated the linear regression analyses with MMSE and age^*^MMSE instead of diagnosis and age^*^diagnosis, the same age effects were found. Details can be found in Supplementary Tables 3, 4.

### Visual ratings per baseline diagnosis and age groups

**Figure 2:**
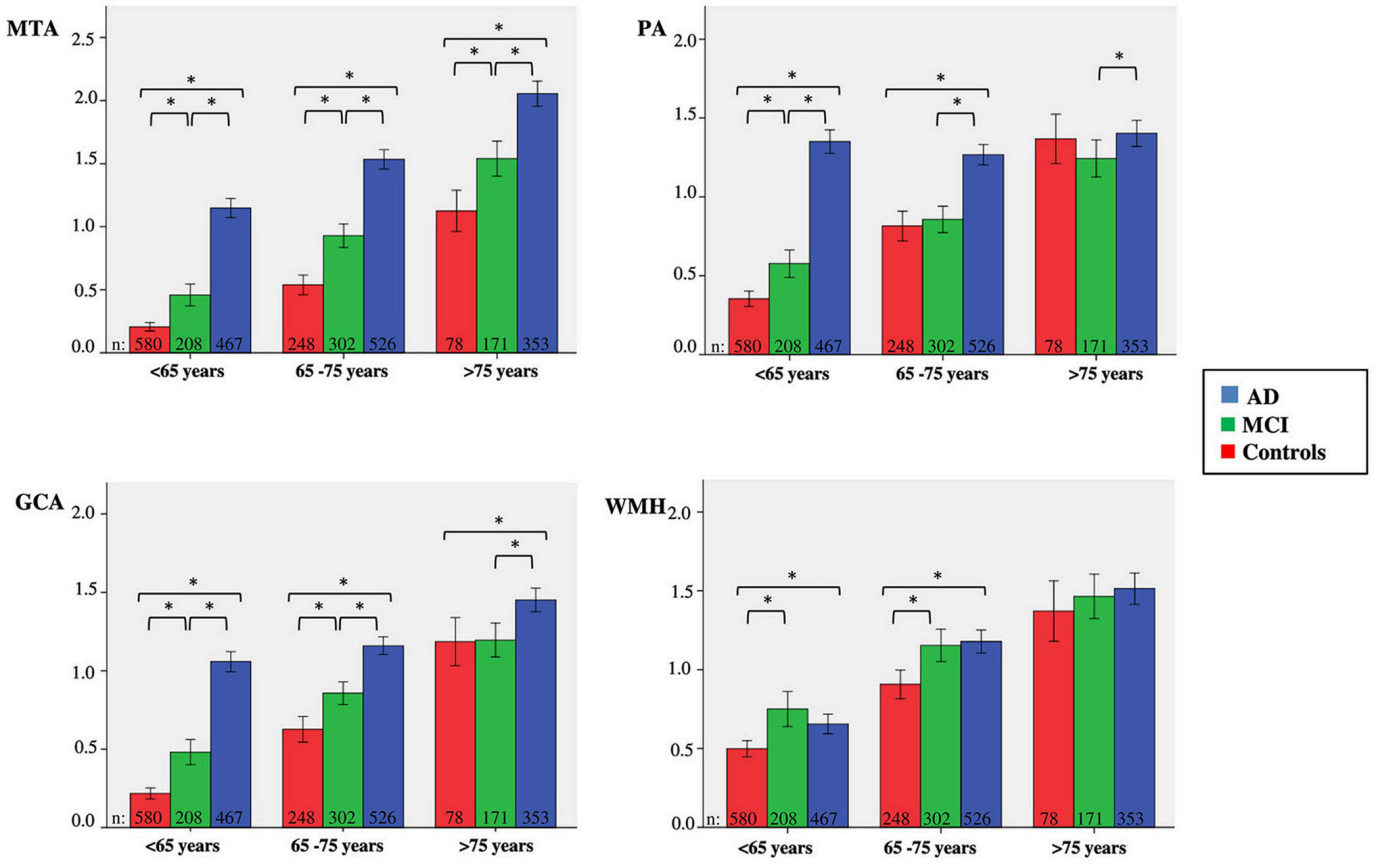
**Clustered bars showing mean MTA, PA, GCA, and WMH scores for age group according to baseline diagnosis**. Y-as: respectively mean MTA (left + right/2), PA (left + right/2), GCA, and WMH, X-as: age group, 95% confidence interval is presented by the error bars, ^*^indicates significant difference between diagnostic groups, using Kruskal-Wallis tests and *post-hoc* Mann-Whitney *U*-tests.

Since there was a clear effect of age on visual ratings, we categorized patients in three age strata; <65 years, 65–75 years and >75 years. Figure 2 visualizes the mean score of each visual rating scale in the different age strata, according to baseline diagnosis. Group sizes for the diagnostic groups by age strata are reported in the figure. For MTA, we found differences between all diagnostic groups in each age group. For PA and GCA, we found differences between all diagnostic groups in <65 years. In addition, for GCA this was also found in the stratum 65–75 years. For PA, in the age group 65–75 years, only AD differed from SCD and MCI, while >75 years AD differed only from MCI. For WMH we found differences between SCD and MCI and between SCD and AD in age groups <65 years and, 65–75 years. There were no differences between diagnostic groups in the >75 years stratum.

### Diagnostic value of visual ratings to separate AD from controls per age group

Based on the highest Youden index, we determined the optimal cut-off for each rating scale in the total group and per age stratum (Table 4). A cut-off of MTA ≥ 1 was optimal for the total group and for <65 years and a cut-off MTA ≥ 1.5 for 65–75 years. In the patients aged >75 years no satisfactory cut-off could be derived. Both PA and GCA add sensitivity in the younger age range, as for these scales we found a high sensitivity at the cost of a lower specificity. A cut-off of an average PA ≥ 1 and GCA ≥ 1 were optimal for <65 years. PA and GCA did not discriminate in the older age groups. WMH did not sufficiently discriminate between groups at all. When we repeated the cross-tabulation for finding the optimal cut-off for MTA in APOE carriers and non-carriers results only changed marginally and optimal cut-offs were comparable (Supplementary Table 5).

**Table 4:** **Discriminatory value of different cut-off points of MTA, PA, GCA, and WMH for differentiating AD from controls in total population and in three age groups**.

**Cut-off point**	**Total *n* = 2253**					**<65 years *n* = 1047**					**65–75 years *n* = 774**					**>75 years *n* = 432**				
	**PPV**	**NPV**	**sens**	**spec**	**Youden**	**PPV**	**NPV**	**Sens**	**Spec**	**Youden**	**PPV**	**NPV**	**Sens**	**Spec**	**Youden**	**PPV**	**NPV**	**Sens**	**Spec**	**Youden**
**MTA**																				
≥0.5	0.76	0.83	0.92	0.58	0.50	0.70	0.85	0.85	0.71	0.56	0.78	0.80	0.95	0.42	0.37	0.83	0.42	0.97	0.10	0.07
≥1	**0.84**	**0.74**	**0.82**	**0.76**	**0.58**	**0.85**	**0.79**	**0.70**	**0.90**	**0.60**	0.82	0.64	0.84	0.62	0.46	0.85	0.46	0.94	0.22	0.16
≥1.5	0.93	0.61	0.60	0.94	0.54	0.96	0.68	0.42	0.98	0.40	**0.94**	**0.53**	**0.61**	**0.92**	**0.53**	0.91	0.42	0.81	0.62	0.43
≥2	0.95	0.52	0.40	0.97	0.37	0.97	0.61	0.21	1.00	0.19	0.96	0.43	0.40	0.96	0.36	0.93	0.32	0.65	0.77	0.42
≥2.5	0.97	0.46	0.21	0.99	0.20	1.00	0.58	0.08	1.00	0.08	0.98	0.37	0.19	0.99	0.18	0.96	0.26	0.41	0.92	0.33
≥3	0.97	0.43	0.11	1.00	0.11	1.00	0.56	0.03	1.00	0.03	1.00	0.34	0.09	1.00	0.09	0.96	0.22	0.25	0.95	0.20
**PA**																				
≥1	0.74	0.73	0.87	0.55	0.42	**0.70**	**0.84**	**0.84**	**0.71**	**0.55**	0.73	0.54	0.87	0.33	0.20	0.82	0.18	0.91	0.09	0.01
≥2	0.87	0.51	0.40	0.91	0.31	0.90	0.67	0.40	0.97	0.37	0.86	0.39	0.36	0.88	0.24	0.86	0.19	0.46	0.58	0.04
≥3	0.87	0.41	0.04	0.99	0.03	0.95	0.56	0.04	1.00	0.04	0.76	0.32	0.03	0.98	0.01	0.89	0.18	0.05	0.97	0.02
**GCA**																				
≥1	**0.78**	**0.75**	**0.86**	**0.64**	**0.50**	**0.75**	**0.82**	**0.79**	**0.79**	**0.58**	0.77	0.61	0.86	0.46	0.32	0.83	0.29	0.93	0.13	0.06
≥2	0.90	0.49	0.34	0.94	0.28	0.98	0.63	0.26	1.00	0.26	0.88	0.38	0.29	0.92	0.21	0.87	0.23	0.51	0.65	0.16
≥3	0.96	0.41	0.02	1.00	0.02	1.00	0.56	0.01	1.00	0.01	0.84	0.32	0.01	1.00	0.01	1.00	0.19	0.04	1.00	0.04
**WMH**																				
≥1	0.67	0.53	0.74	0.45	0.19	0.50	0.61	0.54	0.57	0.11	0.70	0.40	0.80	0.29	0.09	0.82	0.17	0.89	0.10	0.01
≥2	0.77	0.45	0.26	0.88	0.14	0.57	0.56	0.09	0.95	0.06	0.79	0.36	0.31	0.83	0.14	0.83	0.19	0.43	0.60	0.03
≥3	0.85	0.42	0.08	0.98	0.06	0.54	0.56	0.02	0.99	0.01	0.88	0.33	0.08	0.98	0.06	0.89	0.19	0.16	0.91	0.07

*The results are calculated using cross tabulation. Youden index = (sensitivity + specificity) −1. Bold values are the cut-off values that showed the best differentiation*.

*Sens, sensitivity; Spec, specificity; PPV, positive predictive value; NPV, negative predictive value*.

Since MTA, PA, and GCA all had diagnostic value in the age group <65 years, we evaluated if the combination of these scales improved their diagnostic value. Table 5 shows that a combination of MTA with PA or GCA provides a very sensitive and specific indication for AD in the age group <65 years, especially when both ratings are abnormal. In case of one normal and one abnormal rating, the Youden index remained at or below 0.50, and did not add over the application of MTA or GCA/PA alone.

**Table 5:** **Sensitivity and specificity for the combination of MTA and PA and for the combination of MTA and GCA for differentiating AD from controls in age group <65 years**.

	***N***	**Sens**	**Spec**	**Youden**
**COMBINED MTA AND PA:**		***MTA ≥ 1, PA ≥ 1***		
MTA and PA normal	407	*Ref*	*Ref*	*Ref*
MTA normal/PA abnormal	252	0.77	0.72	0.49
MTA abnormal/PA normal	80	0.58	0.92	0.50
MTA and PA abnormal	308	**0.90**	**0.94**	**0.84**
**COMBINED MTA AND GCA:**		***MTA ≥ 1, GCA ≥ 1***		
MTA and GCA normal	465	*Ref*	*Ref*	*Ref*
MTA normal/GCA abnormal	194	0.69	0.81	0.50
MTA abnormal/GCA normal	91	0.57	0.92	0.49
MTA and GCA abnormal	297	**0.86**	**0.94**	**0.80**

*A new variable using 4 levels was created, using only the cut-offs with a Youden index > 0.50 from Table 2. Sensitivity and specificity are calculated using cross tabulation. Youden index = (sensitivity + specificity) −1. Bold values are the combinations that showed the best differentiation. Sens, sensitivity; Spec, specificity*.

### Prediction ability of visual ratings per age group in MCI

Finally, we assessed the predictive value of the visual ratings for dementia due to AD in MCI patients. Details of the demographics and visual ratings of these MCI patients are provided in Table 6. In the patients 65–75 years there was more WMH in the stable MCI as compared to progressive MCI patients, in patients >75 years MTA differed between stable and progressive MCI patients. Results of Cox proportional hazards models are shown in Table 7. Using age-specific cut-offs derived from the controls- AD comparisons, predictive value of MTA was strongest in the oldest MCI patients. PA, GCA, and WMH were not associated with progression to dementia due to AD in any of the age groups. Combination of the visual ratings resulted in a predictive effect for an abnormal MTA with and abnormal PA in the age groups <65 years and 65–75 years. Combination of an abnormal MTA with an abnormal GCA resulted in the same effect in the age group <65 years. When we entered visual rating scales as continuous measures, MTA and WMH were slightly more predictive in older patients, GCA in younger patients. When we repeated the Cox analyses with lower cut-offs only HR of MTA improved slightly in the age group >75 years. When we used progression to any type dementia as outcome measure, results changed only marginally. Details are shown in Supplementary Table 6.

**Table 6:** **Baseline visual ratings of MCI patients according to diagnosis at follow-up by age group**.

		**Stable MCI at FU**	**Progression to AD at FU**
<65 years	N	105	43
	Age	58 ± 5	59 ± 4
	MTA	0.4 ± 0.6	0.5 ± 0.6
	GCA	0.4 ± 0.6	0.6 ± 0.6
	PA	0.5 ± 0.6	0.5 ± 0.5
	WMH	0.7 ± 0.8	0.8 ± 0.8
65–75 years	N	120	79
	Age	70 ± 3	70 ± 3
	MTA	0.8 ± 0.7	0.9 ± 0.8
	GCA	0.7 ± 0.6	0.8 ± 0.6
	PA	0.8 ± 0.7	0.9 ± 0.7
	WMH	1.3 ± 0.9	0.8 ± 0.8^*^
>75 years	N	30	39
	Age	78 ± 2	78 ± 2
	MTA	1.0 ± 0.7	1.7 ± 0.9^*^
	GCA	1.0 ± 0.6	1.2 ± 0.6
	PA	1.2 ± 0.8	1.2 ± 0.7
	WMH	1.3 ± 0.8	1.3 ± 0.9

Values are mean ± standard deviation. Group differences were estimated using Mann- Whitney test. Please note that although we report mean ± standard deviation for the visual rating scales, we used non-parametric tests.

* *Difference between MCI and dementia due to AD at follow up with p < 0.05*.

**Table 7:** **Cox proportional hazard models; influence of MTA, PA, GCA, and WMH and combination of MTA/PA and MTA/GCA on progression of MCI to dementia due to AD in the three age groups**.

	**<65 years *n* = 148**	**65–75 years *n* = 199**	**>75 years *n* = 148**
**MTA**	***MTA ≥ 1***	***MTA ≥ 1.5***	***MTA ≥ 2***
	2.0 (1.0–4.0)	1.3 (0.8–2.2)	**2.2 (1.1–4.6)**
**PA**	***PA ≥ 1***	***PA ≥ 2***	***PA ≥ 2***
	1.6 (0.9–3.0)	1.7 (0.9–3.1)	1.1 (0.5–2.2)
**GCA**	***GCA ≥ 1***	***GCA ≥ 1***	***GCA ≥ 2***
	**2.1 (1.1–3.9)**	1.7 (1.0–2.8)	1.4 (07–3.1)
**WMH**	***WMH ≥ 1***	***WMH ≥ 2***	***WMH ≥ 3***
	1.2 (0.6–2.3)	**0.4 (0.2–0.8)**	2.8 (0.9–8.7)
**MTA AND PA**	***MTA ≥ 1/PA ≥ 1***	***MTA ≥ 1.5/PA ≥ 2***	***MTA ≥ 2/PA ≥ 2***
- MTA and PA normal	*Ref*	*Ref*	*Ref*
- MTA normal/ PA abnormal	1.4 (0.7–2.9)	1.4 (0.7–3.2)	1.1 (0.4–2.6)
- MTA abnormal/ PA normal	1.4 (0.7–2.6)	1.1 (0.8–1.4)	1.5 (0.9–2.2)
- MTA and PA abnormal	**1.3 (1.0–1.8)**	**1.4 (1.0–1.9)**	1.3 (0.9–1.9)
**MTA AND GCA**	***MTA ≥ 1/GCA ≥ 1***	***MTA ≥ 1.5/GCA ≥ 1***	***MTA ≥ 2/GCA ≥ 2***
- MTA and GCA normal	*Ref*	*Ref*	*Ref*
- MTA normal/ GCA abnormal	1.8 (0.8–3.8)	1.5 (0.8–2.6)	1.5 (0.5–4.1)
- MTA abnormal/ GCA normal	1.3 (0.7–2.5)	0.8 (0.4–1.6)	**1.6 (1.0–2.5)**
- MTA and GCA abnormal	**1.5 (1.1–2.0)**	1.2 (1.0–1.6)	1.3 (0.9–1.9)

*Data are presented as hazard ratio (HR) (95% CI). Cox proportional hazard models compared progression to AD with non-converters (= stable MCI at follow-up). Time variable was time to follow-up in years; state variable was progression to AD. The visual ratings were entered dichotomized at the optimal cut-off as was derived from classifying controls from dementia due to AD (Table 2). For the combination of MTA/PA and MTA/GCA a new 4 level variable as presented in Table 3, was used. Sex was entered as co-variate. Bold values are the HR's with p < 0.05*.

## Discussion and conclusion

In this very large memory cohort with a broad age range, we studied the combined effect of age and diagnosis on the visual ratings of atrophy and WMH in controls, MCI and AD. This resulted in three main findings. First, we found an independent effect of age and diagnosis on MTA, resulting in different diagnostic and predictive value in the three age groups. Second, age and diagnosis had a different effect on PA and GCA, providing unequivocal support for their diagnostic value, specifically in younger patients. And third, for WMH we found hardly any diagnostic or predictive value, while this measure was strongly related to age.

Our first finding that MTA is equally affected by age and diagnosis, is consistent with former studies (Launer et al., 1995; Bastos Leite et al., 2004; van de Pol et al., 2006; Barkhof et al., 2007). Earlier studies have suggested age-specific cut-offs for MTA (Scheltens et al., 1992; Koedam et al., 2011; Duara et al., 2013; Pereira et al., 2014; van de Pol and Scheltens, 2014; Ferreira et al., 2015). We found the best diagnostic performance in MTA in the youngest group, with an identified optimal cut-off of MTA ≥ 1, which is the same as the original article but lower than the cut-off of MTA ≥ 1.5 advised by two recent articles (Scheltens et al., 1992, 1995, 1997; Barber et al., 1999; Pereira et al., 2014; Ferreira et al., 2015). Younger subjects should not have medial temporal atrophy at all; at an age <65 years even a MTA score of 1 is suspicious. This finding might be explained by differences in study populations. Our cohort contains a large subgroup <65 years, consisting of 1,047 controls and AD with a mean age of 58 ± 5 years. In former studies assessing the effect of age on MTA, average age of the so-called younger groups was much higher. Also our average MMSE is higher than in most studies, suggesting less advanced disease. The optimal cut-off of MTA ≥ 1.5 for 65–75 years was similar to recent studies (Schoonenboom et al., 2008; Pereira et al., 2014; Ferreira et al., 2015). For the subjects aged >75 years sensitivity and specificity when applying a MTA ≥ 1.5 (sensitivity 0.81; specificity 0.62) or a MTA ≥ 2 (sensitivity 0.65, specificity 0.78) are comparable to previous studies, but the low Youden index indicates that diagnostic performance is modest. When we repeated our linear regression analysis including APOE, we found, comparable to earlier studies, more MTA in APOE carriers and a stronger age effect on MTA in non-carriers (Pereira et al., 2014; Ferreira et al., 2015). Apparently the presence of APOE e4 results in more affected hippocampal region (van der Flier et al., 2011; van de Pol and Scheltens, 2014). The effect of APOE on MTA was subtle however and did not lead to different optimal cut-offs. This is in line with the fact that APOE genotype is generally not used in the diagnostic work-up of AD.

When we attempted to predict progression to AD dementia in patients with MCI, MTA had strongest predictive value in the oldest group >75 years. PA, GCA, and WMH showed no predictive value. In addition, the predictive value of MTA in the younger patients was limited. This was an unexpected result, as previous studies have shown predictive ability for MTA and PA, especially in younger subjects (Korf et al., 2004; Staekenborg et al., 2009; Lehmann et al., 2012, 2013; Prins et al., 2013; Ferreira et al., 2015). However, in our study, the MCI subjects aged <65 years were younger than in previous studies and they had lower MTA scores. In addition, younger patients were less likely to show clinical progression than older patients (<65:28% vs. 65–75:44% vs. >75:56%), resulting in less power. Apparently MCI patients <65 years constitute a different patient category than older MCI patients. It is conceivable that the prototypical patient with MCI due to AD, is a patient that develops a typical, hippocampal type of AD, with an age-at-onset of about 75 years. Younger subjects with the earliest stages of cognitive decline tend to have an atypical presentation, a longer doctors-delay because of misdiagnosis and suffer from a larger penalty on stigmatizing them with MCI (Koedam et al., 2010; Barnes et al., 2015). As a result, younger subjects with AD, often present to a memory clinic already at dementia stage, which may result in a bias for the MCI population in this age group. In older subjects, MCI might be better recognized, which could explain the predictive value of MTA in this group. Also, in the patients 65–75 years there was more WMH in the stable MCI as compared to progressive MCI patients. This suggests that the WMH, rather than AD, could be the cause for their cognitive decline, explaining why this specific group remained stable. Another reason for the low predictive value might be our choice to use the cut-offs derived from controls-AD comparison. One could argue that patients with MCI might have subtler atrophy rates, being earlier in the disease trajectory, thus requiring more sensitive cut-offs. When we repeated the Cox-analyses with lower cut-offs however, predictive values did not improve.

Our second finding concerned the different effects of age and diagnosis on PA and GCA. Previous studies have shown that PA ratings have diagnostic value in early onset AD but do not help the separation of late onset AD from older controls (Koedam et al., 2011; Lehmann et al., 2012, 2013; O'Donovan et al., 2013). To date this has not been reflected by age-specific cut-offs for PA and GCA. To our knowledge, only one study assessed age-specific cut-offs for PA, finding a low diagnostic value, yet advising a cut-off PA ≥1 for all age groups (Ferreira et al., 2015). In our study we found that patients with AD have a high score on PA and GCA regardless of age, while controls and MCI show increased PA and GCA scores with increasing age. These findings resulted in a high diagnostic value for both PA and GCA in patients <65 years, but no value of PA and GCA for patients >65 years. The optimal cut-off for both atrophy measures was a rating of ≥1. The original paper proposed a higher cut-off PA ≥2, resulting in a high specificity at the cost of a low sensitivity (Koedam et al., 2011). With a lower cut-off ≥1 we now found a reverse pattern in the age-group <65 years, with a high sensitivity at the cost of a lower specificity. An additional finding of abnormal MTA greatly adds specificity to PA. In this subgroup of patients <65 years, a combination of an abnormal PA and MTA resulted in very high sensitivity and specificity, hence this should be regarded as alarming. In the preparation of this study, we also used a classification tree to improve the utility of combining visual ratings. However, this tree only added improvement in discriminating controls from AD for both MTA with PA in the age group <65 years. We decided to leave these analyses out of the paper, as the more complex modeling did not add to our message. Furthermore, since our aim was to evaluate the visual ratings as a clinician would, we chose to use as simple as statistics as possible, reflecting clinical practice.

In our study, we found WMH mainly to be affected by age, but not by diagnosis. Various studies have advocated a synergistic effect of SVD and AD pathology on cognitive decline, while other studies have shown that SVD in AD was related to age and vascular risk factors, comparable to individuals without AD (Kester et al., 2014; Mortamais et al., 2014; Spies et al., 2014; Benedictus et al., 2015; Claus et al., 2015; Prins and Scheltens, 2015). Yet, in all these studies the diagnostic value of WMH for separating AD from controls has not been addressed. We found no diagnostic utility for WMH in discriminating AD from controls, which cannot be explained by the relatively young age of our study sample, since even in the oldest age stratum, WMH did not have any discriminatory value. Assessing WMH in the diagnostic work-up remains important, because of the known negative effect of WMH on many outcomes, such as functional decline, (lacunar) infarcts, depression and mortality (Pantoni et al., 2005; van der Flier et al., 2005; Inzitari et al., 2007; Verdelho et al., 2010; Firbank et al., 2012). Furthermore, presence of WMH indicates a possible treatable cause in order to prevent further deterioration (Basile et al., 2006; Prins and Scheltens, 2015). These findings do not oppose the possible interaction of SVD and AD. Since WMH in this study was equally severe in aging controls, one might argue that dementia at older age is by definition “mixed.” Perhaps in older subjects, having WMH, less AD damage is needed to develop dementia (van der Flier et al., 2004; Mortamais et al., 2014). These age effects persisted when we used MMSE score instead of clinical diagnosis which confirms our finding.

These findings have several clinical implications. The value of the visual ratings of atrophy and WMH all differ across the age-groups. This makes it of utmost importance to take into account the age of the patients when using MRI in diagnostic workup. Especially in the younger patients MTA and PA/GCA have diagnostic value; atrophy at an age <65 is a bad sign. By combining MTA with PA/GCA, the value even increases. Older age reduces the value of rating scales substantially, in older patients it is harder to separate age-effect from AD- effect. These findings are in line with the classical Braak model for MTA (Braak et al., 2006). However, the findings for PA are not in line with Braak, since especially young subjects showed severe PA only in AD cases, which is not observed in controls and MCI, whereas this difference disappears in increasing age. This suggests a separate pathological stageing-model for younger patients may be warranted (Jagust et al., 2008; Fjell et al., 2013). In this patient group, the use of visual ratings should be used to rule-out AD in case of no atrophy rather than proving inclusive evidence for AD when there is atrophy. Perhaps in the future more automated measures will be able to distinguish pathological from age-adequate brain aging, being able to pick up more subtle effects (Koikkalainen et al., 2016). Automatic quantification methods of brain atrophy, and other modalities such as FDG-PET, also have the advantage of providing objective measures, independent of the expertise of the clinician, whereas visual ratings are a subjective visual interpretation. Furthermore, these automatic methods are able to extract more information and combine information, for example on WMH and atrophy, and provide an estimate of the underlying neurodegenerative disease. Visual rating of MRI's have the advantage however that they are more feasible in daily clinical practice. Automatic quantification methods are dependent on scan protocol and quality, whereas visual ratings can be applied to images acquired with less advanced scanners. Also these automatic methods often require costly and time-consuming software-programs, while visual ratings can be applied in an instant, with the patient in front of the clinician.

This study has several limitations. First, the lack of neuropathological confirmation of diagnosis. Especially in elderly patients, with comorbid SVD, atrophy might also be the result of WMH or hippocampal sclerosis and not of amyloid pathology (Barkhof et al., 2007). Due to this we might have selected patients that have been misclassified with AD. However, in this study we found a similar degree of WMH in all elderly subjects, regardless of diagnosis, diminishing the importance of specifying the etiology as mixed or not. Second, we used SCD as controls, although we cannot exclude the possibility that these patients had underlying AD. We feel that the comparison of AD with SCD patients is a clinically relevant comparison however, as this is the differential diagnosis that a clinician has to make every day. Furthermore, underlying AD can also not be excluded in “pure” controls, as it is known that roughly one third of normal elderly harbors AD pathology (Chetelat et al., 2010; Vos et al., 2013). Third, the mean follow-up of 2.5 ± 1.7 years could imply that MCI patients, who remained stable during this period, might still progress to dementia after longer follow up. Fourth, in our clinical work-up clinicians are not blinded for the MRI results. This might have resulted in bias. The effect of the MRI results on diagnosis might have also changed throughout the time due to changing insights in use of biomarkers. However, all diagnoses were made in our multidisciplinary consensus meeting, in which the clinical characteristics of the patient and the cognitive profile on neuropsychological testing is leading. A final limitation could be the use of different scanners with increasing field strength throughout the time. This could also be regarded as a strength however, as the visual ratings have the advantage that they are robust for scanner differences and easy to use.

Among the strengths of the current study is our harmonized diagnostic protocol according to which all patients were analyzed. All patients were selected from the same memory clinic. The large sample size and the broad age spectrum ranging from 45 to 95 makes these results robust. Furthermore, the scans were rated by experienced researchers after they had completed the required training (van der Flier et al., 2014).

To conclude, visual ratings are of use in daily practice, but should be interpreted with caution and with reference to a patients' age. The current research criteria advise the use of MTA in the diagnostic work-up for AD, but do not specify the amount of atrophy or the effect of age (Dubois et al., 2007, 2014). This study shows that MTA is strongly influenced by age and that age related cut-offs are needed. PA and GCA seem to be of equal use for the diagnostic workup in patients <65 years, and their information is incremental to the information in the MTA scale. Taking into account age-specific cut-offs and characteristics of each visual rating scale, use of visual rating scales for MRI can enhance recognition of AD for either diagnostic or research purposes, especially in younger patients.

## Ethics statement

This study was carried out in accordance with the recommendations of the medical ethical committee of the VU Medical Center with written informed consent from all subjects. All subjects gave written informed consent in accordance with the Declaration of Helsinki. The protocol was approved by the medical ethical committee of the VU Medical Center.

## Author contributions

HR drafted the manuscript and analyzed/interpreted data. MB, MW, FB, PS, and MM revised the manuscript and interpreted the data. WF drafted the manuscript, analyzed/interpreted data and supervised the project.

### Conflict of interest statement

MW received speaking and consultancy fees from Biogen, Novartis, and Roche. FB serves/has served on the advisory boards of Bayer-Schering Pharma, Sanofi-Aventis, Biogen-Idec, TEVA, Merck-Serono, Novartis, Roche, Synthon BV, Jansen Research and Genzyme. He received funding from the Dutch MS Society and EU-FP7 and has been a speaker at symposia organised by the Serono Symposia Foundation and MedScape. PS has served as consultant for Wyeth-Elan, Genentech, Danone and Novartis and received funding for travel from Pfizer, Elan, Janssen, and Danone Research. WF performs contract research for Boehringer Ingelheim. Research programs of WF have been funded by ZonMW, NWO, EU-FP7, Alzheimer Nederland, CardioVascular Onderzoek Nederland, stichting Dioraphte, Gieskes-Strijbis fonds, Boehringer Ingelheim, Piramal Neuroimaging, Roche BV, Janssen Stellar. All funding is paid to her institution. HR, MB, and MM declare that the research was conducted in the absence of any commercial or financial relationship that could be construed as a potential conflict of interest.
