# Supplementary material for: MRI Visual Ratings of Brain Atrophy and White Matter Hyperintensities across the Spectrum of Cognitive Decline Are Differently Affected by Age and Diagnosis
Source: Front Aging Neurosci. 2017 May 9;9:117. doi: 10.3389/fnagi.2017.00117 (PMC5422528; doi:10.3389/fnagi.2017.00117)
Supplement: Supplementary file 1 [file Table1.docx]

Supplementary Material

The combined effect of age and diagnosis on MRI visual ratings in MCI and AD in large memory cohort

**Hanneke FM Rhodius- Meester*, Marije R Benedictus, Mike P Wattjes, Frederik Barkhof, Philip Scheltens, Majon Muller, Wiesje M van der Flier**

*** Correspondence:** Corresponding author: h.rhodius@vumc.nl

**Supplementary table 1** Acquisition parameters per scanner used.

TR: repetition time in milliseconds, TE: echo time in milliseconds, TI: inversion time in milliseconds, FA: flip angle, voxel size in millimeters.

| **Scanner** |  |  | **Plane** | **TR** | **TE** | **TI** | **FA** | **Voxel size** |
| --- | --- | --- | --- | --- | --- | --- | --- | --- |
| **Avanto** | **1.5T** | **T1** | 3D coronal | 2700 | 5 | 950 | 8° | 1×1×1.50 |
|  |  | **Flair** | 2D transversal | 9000 | 89 | 2500 | 150° | 0.45×0.45×5 |
|  |  |  |  |  |  |  |  |  |
| **Discovery** | **3T** | **T1** | 3D sagittal | 8 | 3 | 450 | 12° | 0.98×0.98×1 |
|  |  | **Flair** | 3D sagittal | 8000 | 129 | 2341 | 90° | 0.98×0.98×1 |
|  |  |  |  |  |  |  |  |  |
| **Impact** | **1T** | **T1** | 3D coronal | 15 | 7 | 300 | 15° | 1×1×1.50 |
|  |  | **Flair** | 2D transversal | 9000 | 105 | 2200 | 180° | 0.98×0.98×5 |
|  |  |  |  |  |  |  |  |  |
| **PETMR** | **3T** | **T1** | 3D sagittal | 8 | 4 | - | 8° | 1×1×1 |
|  |  | **Flair** | 3D sagittal | 4800 | 278 | 1650 | 90° | 1.04×1.04×1.12 |
|  |  |  |  |  |  |  |  |  |
| **Signa** | **1.5T** | **T1** | 3D sagittal | 12 | 5 | 450 | 12° | 0.98×0.98×1.5 |
|  |  | **Flair** | 3D sagittal | 6500 | 117 | 1988 | 90° | 1.2×1.2×1.3 |
|  |  |  |  |  |  |  |  |  |
| **Signa** | **3T** | **T1** | 3D sagittal | 8 | 3 | 450 | 12° | 0.98×0.98×1 |
|  |  | **Flair** | 3D sagittal | 8000 | 126 | 2347 | 90° | 0.98×0.98×1.2 |
|  |  |  |  |  |  |  |  |  |
| **Sonata** | **1.5T** | **T1** | 3D coronal | 2400 | 5 | 1000 | 6° | 1×1×1.5 |
|  |  | **Flair** | 2D transversal | 9000 | 108 | 2500 | 150° | 0.98×0.98×6 |
|  |  |  |  |  |  |  |  |  |
| **Titan** | **3T** | **T1** | 3D sagittal | 9 | 3 | 800 | 7° | 1×1×1 |
|  |  | **Flair** | 3D sagittal | 6000 | 401 | 2000 | 90° | 0.49×0.49×2 |
|  |  |  |  |  |  |  |  |  |
| **Vision** | **1.5T** | **T1** | 3D coronal | 15 | 7 | - | 8° | 0.98×0.98×1.5 |
|  |  | **Flair** | 2D transversal | 9000 | 105 | 2200 | 180° | 0.90×0.90×5 |
