# Supplementary material for: MRI Visual Ratings of Brain Atrophy and White Matter Hyperintensities across the Spectrum of Cognitive Decline Are Differently Affected by Age and Diagnosis
Source: Front Aging Neurosci. 2017 May 9;9:117. doi: 10.3389/fnagi.2017.00117 (PMC5422528; doi:10.3389/fnagi.2017.00117)
Supplement: Supplementary file 2 [file Table2.docx]

Supplementary Material

The combined effect of age and diagnosis on MRI visual ratings in MCI and AD in large memory cohort

**Hanneke FM Rhodius- Meester*, Marije R Benedictus, Mike P Wattjes, Frederik Barkhof, Philip Scheltens, Majon Muller, Wiesje M van der Flier**

*** Correspondence:** Corresponding author: h.rhodius@vumc.nl

**Supplementary table 2** Correlation matrix using Spearmans rho for the total group.

* Correlation is significant at the 0.01 level (two tailed)

|  | **MTA** | **PA** | **GCA** | **WMH** |
| --- | --- | --- | --- | --- |
| **MTA** | 1.00 | .414* | .549* | .337* |
| **PA** | .414* | 1.00 | .732* | .133* |
| **GCA** | .549* | .732* | 1.00 | .256* |
| **WMH** | .337* | .133* | .256* | 1.00 |
