# Supplementary material for: MRI Visual Ratings of Brain Atrophy and White Matter Hyperintensities across the Spectrum of Cognitive Decline Are Differently Affected by Age and Diagnosis
Source: Front Aging Neurosci. 2017 May 9;9:117. doi: 10.3389/fnagi.2017.00117 (PMC5422528; doi:10.3389/fnagi.2017.00117)
Supplement: Supplementary file 4 [file Table4.docx]

Supplementary Material

The combined effect of age and diagnosis on MRI visual ratings in MCI and AD in large memory cohort

**Hanneke FM Rhodius- Meester*, Marije R Benedictus, Mike P Wattjes, Frederik Barkhof, Philip Scheltens, Majon Muller, Wiesje M van der Flier**

*** Correspondence:** Corresponding author: h.rhodius@vumc.nl

**Supplementary table 4** Combined effect of age and MMSE on visual ratings.

Linear regression analyses were used, using separate models for each rating scale. As independent variables we entered MMSE (continous), age (continuous) and the interaction term for age*MMSE. St beta: standardized coefficients beta, p: p value, F: Fisher, df: degrees of freedom

|  | **MTA** | |  | **PA** | |  | **GCA** | |  | **WMH** | |
| --- | --- | --- | --- | --- | --- | --- | --- | --- | --- | --- | --- |
|  | **St beta** | **p** |  | **St beta** | **p** |  | **St beta** | **p** |  | **St beta** | **p** |
| Constant | -.74 | .120 |  | 4.23 | <.001 |  | 2.06 | <.001 |  | -1.74 | .001 |
| Sex, male | .08 | <.001 |  | .08 | <.001 |  | .09 | <.001 |  | -.05 | .002 |
| Age | .50 | <.001 |  | -.39 | <.001 |  | -.04 | .620 |  | .50 | <.001 |
| **Cognition** |  |  |  |  |  |  |  |  |  |  |  |
| MMSE | -.31 | .003 |  | -1.36 | <.001 |  | -.98 | <.001 |  | .05 | .669 |
| **Interaction** |  |  |  |  |  |  |  |  |  |  |  |
| Age*MMSE | -.07 | .555 |  | 1.21 | <.001 |  | .77 | <.001 |  | -.14 | .301 |
|  |  |  |  |  |  |  |  |  |  |  |  |
| R square | .39 | |  | .23 | |  | .30 | |  | .19 | |
| F | 473.85 | |  | 215.79 | |  | 314.37 | |  | 173.75 | |
| df regression | 4 |  |  | 4 |  |  | 4 |  |  | 4 |  |
| df residual | 2929 |  |  | 2928 |  |  | 2929 |  |  | 2929 |  |
