# Supplementary material for: MRI Visual Ratings of Brain Atrophy and White Matter Hyperintensities across the Spectrum of Cognitive Decline Are Differently Affected by Age and Diagnosis
Source: Front Aging Neurosci. 2017 May 9;9:117. doi: 10.3389/fnagi.2017.00117 (PMC5422528; doi:10.3389/fnagi.2017.00117)
Supplement: Supplementary file 5 [file Table5.docx]

Supplementary Material

The combined effect of age and diagnosis on MRI visual ratings in MCI and AD in large memory cohort

**Hanneke FM Rhodius- Meester*, Marije R Benedictus, Mike P Wattjes, Frederik Barkhof, Philip Scheltens, Majon Muller, Wiesje M van der Flier**

*** Correspondence:** Corresponding author: h.rhodius@vumc.nl

**Supplementary table 5** Discriminatory value for different cut-off points of MTA for differentiation AD from controls in total population and in three age groups, stratified for APOE e4 carrier and non-carrier.

The results are calculated using cross tabulation. Youden index= (sensitivity+ specificity)-1. Highlighted are the cut-off values that showed the best differentiation.

Sens: sensitivity, Spec: specificity, PPV: positive predictive value, NPV: negative predictive value.

| **cut- off point** | | **Total** | | | | |  | **<65 years** | | | | |  | **65-75 years** | | | | |  | **>75 years** | | | | |
| --- | --- | --- | --- | --- | --- | --- | --- | --- | --- | --- | --- | --- | --- | --- | --- | --- | --- | --- | --- | --- | --- | --- | --- | --- |
|  |  | **PPV** | **NPV** | **sens** | **spec** | **Youden** |  | **PPV** | **NPV** | **Sens** | **Spec** | **Youden** |  | **PPV** | **NPV** | **Sens** | **Spec** | **Youden** |  | **PPV** | **NPV** | **Sens** | **Spec** | **Youden** |
| **APOE carriers** | | |  |  |  |  |  |  |  |  | | |  |  |  |  | | |  |  |  |  | | |
| **MTA:** |  | n=1010 (281 controls, 729 AD) | | | | |  | n=474 (188 controls, 286 AD) | | | | |  | n=404(79 controls, 325 AD) | | | | |  | n=132 (14 controls, 118 AD) | | | | |
| ≥ 0.5 |  | 0.84 | 0.71 | 0.92 | 0.54 | 0.46 |  | 0.78 | 0.76 | 0.87 | 0.63 | 0.50 |  | 0.87 | 0.65 | 0.95 | 0.39 | 0.34 |  | 0.90 | 0.25 | 0.95 | 0.14 | 0.09 |
| ≥ 1 |  | **0.90** | **0.60** | **0.81** | **0.76** | **0.57** |  | **0.88** | **0.65** | **0.70** | **0.85** | **0.55** |  | **0.91** | **0.52** | **0.85** | **0.65** | **0.50** |  | 0.92 | 0.27 | 0.91 | 0.28 | 0.19 |
| ≥ 1.5 |  | 0.97 | 0.46 | 0.56 | 0.95 | 0.51 |  | 0.97 | 0.53 | 0.43 | 0.98 | 0.41 |  | **0.96** | **0.36** | **0.61** | **0.89** | **0.50** |  | **0.98** | **0.32** | **0.78** | **0.86** | **0.64** |
| ≥ 2 |  | 0.98 | 0.37 | 0.34 | 0.98 | 0.32 |  | 0.97 | 0.45 | 0.21 | 0.99 | 0.20 |  | 0.98 | 0.27 | 0.38 | 0.96 | 0.34 |  | 0.99 | 0.21 | 0.58 | 0.93 | 0.51 |
| ≥ 2.5 |  | 0.98 | 0.31 | 0.17 | 0.99 | 0.16 |  | 1.00 | 0.42 | 0.07 | 1.00 | 0.07 |  | 0.98 | 0.22 | 0.17 | 0.99 | 0.16 |  | 0.98 | 0.15 | 0.38 | 0.93 | 0.31 |
| ≥ 3 |  | 0.98 | 0.30 | 0.08 | 1.00 | 0.08 |  | 1.00 | 0.40 | 0.002 | 1.00 | 0.02 |  | 1.00 | 0.21 | 0.08 | 1.00 | 0.08 |  | 0.97 | 0.13 | 0.24 | 0.93 | 0.17 |
| **APOE non-carriers** | | | |  |  |  |  |  |  |  | | |  |  |  |  | | |  |  |  |  | | |
| **MTA:** |  | n=869 (507 controls, 362 AD) | | | | |  | n=485 (339 controls, 146 AD) | | | | |  | n=289 (151 controls, 138 AD) | | | | |  | n=95 (17 controls, 78 AD) | | | | |
| ≥ 0.5 |  | 0.65 | 0.91 | 0.91 | 0.65 | 0.56 |  | 0.61 | 0.91 | 0.83 | 0.77 | 0.60 |  | 0.62 | 0.93 | 0.96 | 0.45 | 0.41 |  | 0.83 | 0.33 | 0.95 | 0.12 | 0.07 |
| ≥ 1 |  | **0.74** | **0.84** | **0.78** | **0.81** | **0.59** |  | **0.78** | **0.87** | **0.69** | **0.92** | **0.61** |  | 0.66 | 0.76 | 0.78 | 0.63 | 0.41 |  | 0.85 | 0.44 | 0.93 | 0.24 | 0.17 |
| ≥ 1.5 |  | 0.92 | 0.75 | 0.54 | 0.97 | 0.51 |  | 0.95 | 0.76 | 0.39 | 0.99 | 0.38 |  | **0.91** | **0.71** | **0.58** | **0.95** | **0.53** |  | 0.90 | 0.36 | 0.77 | 0.59 | 0.36 |
| ≥ 2 |  | 0.94 | 0.68 | 0.36 | 0.98 | 0.35 |  | 0.96 | 0.74 | 0.18 | 1.00 | 0.18 |  | 0.94 | 0.65 | 0.42 | 0.97 | 0.39 |  | 0.92 | 0.30 | 0.62 | 0.76 | 0.38 |
| ≥ 2.5 |  | 0.99 | 0.63 | 0.18 | 1.00 | 0.18 |  | 1.00 | 0.71 | 0.06 | 1.00 | 0.06 |  | 0.97 | 0.58 | 0.20 | 0.99 | 0.19 |  | 1.00 | 0.26 | 0.37 | 1.00 | 0.37 |
| ≥ 3 |  | 1.00 | 0.60 | 0.08 | 1.00 | 0.08 |  | 1.00 | 0.70 | 0.04 | 1.00 | 0.04 |  | 1.00 | 0.54 | 0.07 | 1.00 | 0.07 |  | 1.00 | 0.22 | 0.22 | 1.00 | 0.22 |
