# Supplementary material for: MRI Visual Ratings of Brain Atrophy and White Matter Hyperintensities across the Spectrum of Cognitive Decline Are Differently Affected by Age and Diagnosis
Source: Front Aging Neurosci. 2017 May 9;9:117. doi: 10.3389/fnagi.2017.00117 (PMC5422528; doi:10.3389/fnagi.2017.00117)
Supplement: Supplementary file 6 [file Table6.docx]

Supplementary Material

The combined effect of age and diagnosis on MRI visual ratings in MCI and AD in large memory cohort

**Hanneke FM Rhodius- Meester*, Marije R Benedictus, Mike P Wattjes, Frederik Barkhof, Philip Scheltens, Majon Muller, Wiesje M van der Flier**

*** Correspondence:** Corresponding author: h.rhodius@vumc.nl

**Supplementary table 6** Cox proportional hazard models; influence of MTA, PA, GCA and WMH on progression of MCI to dementia or AD in the three age groups

Data are presented as hazard ratio (HR) (95% CI). Cox proportional hazard models compared progression to AD and progression to all dementias with nonconverters (= stable MCI at follow- up). Time variable was time to follow-up in years; state variable was progression to AD and to dementia. The visual ratings were entered in separate models as continuous variables and dichotomized at the optimal cut-off as was derived from classifying controls from dementia due to AD (table 2). Sex was entered as co-variate. Highlighted are the HR’s with p <.05. a: data also shown in table 5.

|  | **<65 years** |  | **65-75 years** |  | **>75 years** |
| --- | --- | --- | --- | --- | --- |
|  |  |  |  |  |  |
| **MTA continuous** |  |  |  |  |  |
| - Progression to AD | 1.3 (0.8-2.2) |  | **1.4 (1.0-1.9)** |  | **1.6 (1.2-2.2)** |
| - Progression to dementia | 1.4 (0.9-1.9) |  | **1.4 (1.1-1.9)** |  | **1.6 (1.2-2.1)** |
| **MTA ≥0.5** |  |  |  |  |  |
| - Progression to AD | 1.3 (0.7-2.5) |  | 1.5 (0.9-2.6) |  | 3.8 (0.5-28.2) |
| - Progression to dementia | 1.4 (0.8-2.4) |  | 1.5 (0.9-2.4) |  | 4.1 (0.6-30.8) |
| **MTA ≥1** |  |  |  |  |  |
| - Progression to AD | 2.0 (1.0-4.0)^a^ |  | 1.3 (0.8-2.1) |  | **4.8 (1.4-16.1)** |
| - Progression to dementia | **2.0 (1.1-3.5)** |  | 1.3 (0.9-2.0) |  | **5.0 (1.6-16.9)** |
| **MTA ≥1.5** |  |  |  |  |  |
| - Progression to AD | 0.9 (0.-4.0) |  | 1.3 (0.8-2.2)^a^ |  | **2.7 (1.3-5.5)** |
| - Progression to dementia | 1.2 (0.5-2.9) |  | 1.2 (0.9-2.2) |  | **2.5 (1.3-4.7)** |
| **MTA ≥2** |  |  |  |  |  |
| - Progression to AD | 1.5 (0.4-6.7) |  | 1.3 (0.7-2.5) |  | **2.2 (1.1-4.6)**^a^ |
| - Progression to dementia | 1.7 (0.6-4.7) |  | 1.5 (0.9-2.5) |  | **2.1 (1.1-3.9)** |
| **MTA ≥2.5** |  |  |  |  |  |
| - Progression to AD | 0.7 (0.1-5.6) |  | **3.9 (2.9-16.6)** |  | 1.9 (0.8-4.1) |
| - Progression to dementia | 1.3 (0.4-4.2) |  | **4.9 (2.5-9.5)** |  | 1.6 (0.8-3.4) |
| **MTA ≥3** |  |  |  |  |  |
| - Progression to AD | *no subjects with* |  | **5.9 (1.4-25.0)** |  | 1.8 (0.7-4.6) |
| - Progression to dementia | *MTA ≥3* |  | **3.7 (1.5-9.2)** |  | 1.6 (0.6-4.1) |
| **PA continuous** |  |  |  |  |  |
| - Progression to AD | 1.8 (1.0-3.3) |  | 1.4 (1.0-2.0) |  | 1.2 (0.8-1.9) |
| - Progression to dementia | **1.9 (1.2-3.1)** |  | 1.3 (0.9-1.7) |  | 1.2 (0.8-1.8) |
| **PA ≥1** |  |  |  |  |  |
| - Progression to AD | 1.6 (0.9-3.0)^a^ |  | 1.4 (0.8-2.2) |  | 1.3 (0.5-3.6) |
| - Progression to dementia | **1.8 (1.1-3.1)** |  | 1.3 (0.8-1.9) |  | 1.1 (0.5-2.6) |
| **PA ≥2** |  |  |  |  |  |
| - Progression to AD | 7.4 (0.9-61.0) |  | 1.7 (0.9-3.1)^a^ |  | 1.1 (0.5-2.2)^a^ |
| - Progression to dementia | **4.0 (1.2-13.3)** |  | 1.6 (0.9-2.8) |  | 1.2 (0.7-2.3) |
| **PA ≥3** |  |  |  |  |  |
| - Progression to AD | *no subjects with* |  | *no subjects with* |  | *no subjects with* |
| - Progression to dementia | *PA ≥3* |  | *PA ≥3* |  | *PA ≥3* |
| **GCA continuous** |  |  |  |  |  |
| - Progression to AD | **2.0 (1.3-4.5)** |  | 1.4 (0.9-2.0) |  | 1.7 (1.0-3.0) |
| - Progression to dementia | **1.8 (1.2-2.8)** |  | 1.4 (1.0-1.9) |  | 1.7 (1.0-2.7) |
| **GCA ≥1** |  |  |  |  |  |
| - Progression to AD | **2.1 (1.1-3.9)**^a^ |  | 1.7 (1.0-2.8)^a^ |  | 3.2 (0.9-10.7) |
| - Progression to dementia | **1.8 (1.1-3.0)** |  | **1.6 (1.0-2.6)** |  | 2.2 (0.8-5.6) |
| **GCA ≥2** |  |  |  |  |  |
| - Progression to AD | **4.5 (1.4-15.0)** |  | 1.1 (0.5-2.5) |  | 1.4 (07-3.1)^a^ |
| - Progression to dementia | **3.4 (1.2-9.6)** |  | 1.3 (0.7-2.4) |  | 1.5 (0.8-3.0) |
| **GCA ≥3** |  |  |  |  |  |
| - Progression to AD | *no subjects with* |  | *no subjects with* |  | *no subjects with* |
| - Progression to dementia | *GCA ≥3* |  | *GCA ≥3* |  | *GCA ≥3* |
| **WMH continuous** |  |  |  |  |  |
| - Progression to AD | 0.9 (0.6-1.2) |  | **0.8 (0.6-1.0)** |  | **1.6 (1.1-2.5)** |
| - Progression to dementia | 1.0 (0.8-1.4) |  | 0.9 (0.7-1.1) |  | **1.8 (1.2-2.6)** |
| **WMH ≥1** |  |  |  |  |  |
| - Progression to AD | 1.2 (0.6-2.3)^a^ |  | 0.8 (0.5-1.3) |  | 1.9 (0.7-5.1) |
| - Progression to dementia | 1.4 (0.8-2.4) |  | 1.0 (0.6-1.6) |  | 2.2 (0.8-5.6) |
| **WMH ≥2** |  |  |  |  |  |
| - Progression to AD | 0.4 (0.1-1.2) |  | **0.4 (0.2-0.8)**^a^ |  | 1.9 (0.9-3.9) |
| - Progression to dementia | 0.9 (0.4-1.8) |  | 0.6 (0.4-1.0) |  | **2.0 (1.1-3.7)** |
| **WMH ≥3** |  |  |  |  |  |
| - Progression to AD | 0.4 (0.1-1.8) |  | 0.5 (0.2-1.7) |  | 2.8 (0.9-8.7)^a^ |
| - Progression to dementia | 0.7 (0.3-2.0) |  | 0.6 (0.2-1.5) |  | **3.8 (1.5-9.6)** |
